# Enantioselective Oxidative Stress and DNA Damage Induced by *Rac*- and *S*-metolachlor on the Earthworm *Eisenia fetida*

**DOI:** 10.3390/toxics11030246

**Published:** 2023-03-06

**Authors:** Yong Yang, Li Li, Zhaozhen Luo, Yuqiang Zhao, Yalin Mu, Qingming Zhang

**Affiliations:** 1 Shandong Engineering Research Center for Environment-Friendly Agricultural Pest Management, College of Plant Health and Medicine, Qingdao Agricultural University, Qingdao 266109, China; 2 Comprehensive Law Enforcement Team of Ecological Environment Protection, Rizhao Bureau of Ecological Environment, Rizhao 276826, China; 3 Junan County Agriculture and Rural Bureau, Linyi 276600, China; 4 Junan County Wanghailou State-owned Forest Farm, Linyi 276600, China

**Keywords:** chiral pesticide, enantioselectivity, earthworm, oxidative stress, genotoxicity, degradation

## Abstract

Metolachlor is a widely used chiral herbicide. However, information on its enantioselective toxicity to earthworms, an important soil organism, remains limited. Herein, the effects of *Rac*- and *S*-metolachlor on oxidative stress and DNA damage in *Eisenia fetida* were investigated and compared. Moreover, the degradation of both herbicides in the soil was also determined. The results showed that reactive oxygen species (ROS) in *E. fetida* were more easily induced by *Rac*-metolachlor than *S*-metolachlor at a higher concentration (above 16 µg/g). Similarly, the effects of *Rac*-metolachlor on superoxide dismutase (SOD) activity and DNA damage in *E. fetida* were more significant than those of *S*-metolachlor at the same exposure concentration and time. *Rac*- and *S*-metolachlor did not result in severe lipid peroxidation. The toxic effects of both herbicides on *E. fetida* gradually decreased after 7 days as the exposure was prolonged. At the same concentration, *S*-metolachlor degrades faster than *Rac*-metolachlor. These results suggest that *Rac*-metolachlor has a greater effect on *E. fetida* than *S*-metolachlor, providing a significant reference for the rational use of metolachlor.

## 1. Introduction

Pesticides are currently playing a vital role in crop health and food safety in agriculture worldwide. Among all pesticides, chiral pesticides are a special class that commonly has one or more chiral centers [1]. The proportion of chiral pesticides has gradually increased for the last decades and, at present, about 30% of commercial pesticides are chiral compounds [2]. The enantiomers of these chiral pesticides have identical physicochemical properties but they may exhibit different biological activity, toxicological effects, and ecological behavior [3]. Therefore, elucidating the differences in the enantiomers of chiral pesticides is essential for the development of highly efficient and environmentally friendly green pesticides.

Metolachlor [(aRS,1RS)-2-chloro-60-ethyl-N-(2-methoxy-1-methylethyl) acet-o-toluidide] is a chloroacetanilide herbicide widely used in corn, soybean, and other dry crops to control pre-emergent broadleaf and grass weeds. The structure of metolachlor contains two chiral centers (a chiral carbon and a chiral axis) and thus has four stereoisomers: aS,1S-, aR,1S-, aS,1R-, and aR,1R-. There are two commercial herbicides currently on the market: one is a racemate (*Rac*-metolachlor), containing an equal ratio of *R* and *S* isomer, and the other is *S*-metolachlor enriched with high-efficiency S isomer. Compared to *Rac*-metolachlor, the herbicidal activity of *S*-metolachlor increased by 1.6 times [1,2]. In China, metolachlor is an important herbicide for controlling preemergent broadleaf weeds. By 2022, there was 170 registration information for *Rac*-metolachlor (including technical material and pesticide preparation), and 83 registration information for *S*-metolachlor were retrieved through the China Pesticide Information Network (http://www.icama.org.cn/hysj/index.jhtml, 31 December 2022). Metolachlor has a high water solubility (530 mg/L) and is easily detected in a water environment [3]. It has been shown that metolachlor can produce enantiomeric selective bioaccumulation and toxic effects on aquatic organisms. Previous studies reported that *Rac*-metolachlor has stronger toxic effects on hydrophyte microalgae *Chlorella pyrenoidosa* than *S*-metolachlor [4], while another microalga, *Scenedesmus obliquus*, is more sensitive to *S*-metolachlor than *Rac*-metolachlor [5]. Metolachlor also has an enantioselectivity effect on fish. For instance, the latest research conducted by Ou-Yang et al. reported that *S*-metolachlor was preferentially accumulated in zebrafish (*Danio rerio*) at low concentrations, while *Rac*-metolachlor was preferentially enriched at high concentrations. Moreover, compared with *S*-metolachlor, *Rac*-metolachlor with high concentrations show greater toxic effects on antioxidant enzyme activities, liver development, and endocrine disturbance in zebrafish [6]. On soil organisms, the study by Xu et al. [7] showed that *Rac*-metolachlor has a higher toxic effect on avoidance behavior, body weight change, and cellulase activity of an earthworm (*Eisenia fetida*) than those of *S*-metolachlor at the same concentrations. However, the enantiomeric selective effects of metolachlor on oxidative stress and genetic toxicity in earthworms remain limited.

In this study, therefore, we further evaluated the toxicity differences of *Rac*-metolachlor and *S*-metolachlor on the earthworm *E. fetida* in terms of antioxidant enzyme levels and DNA damage. We also determined the residue amount of *Rac*-metolachlor and *S*-metolachlor in soils containing *E. fetida*. The results will further supply essential information for an adequate understanding of the ecotoxic differences between *Rac*-metolachlor and *S*-metolachlor, and will help guide the rational use of metolachlor.

## 2. Materials and Methods

### 2.1. Chemicals and Reagents

*Rac*-metolachlor (95% purity) and *S*-metolachlor (96% purity) were obtained from Binnong Technology Co., Ltd., Binzhou, China. The reagent kits for assaying superoxide dismutase (SOD) and malondialdehyde (MDA) were purchased from Nanjing Jiancheng Bioengineering Institute, China. All other reagents used were HPLC-grade or analytical-grade purity.

### 2.2. Earthworm and Soil

The earthworms with obvious clitellum used in this study were *E. fetida* (300–500 mg bodyweight), which were purchased from Wangjun Earthworm Breeding Farm (Jurong, Jiangsu province, China). The earthworms were first acclimatized for one week in an artificial climate incubator (Jiangnan, Ningbo, China, 20 ± 1 °C) prior to tests. The soil used in this study was artificial soil. The artificial soil was made by mixing a ratio of 70% silica sand, 20% kaolin clay, and 10% sphagnum peat moss, and the pH was adjusted to 6.5 ± 0.5 by mixing CaCO_3_ [8].

### 2.3. Exposure Experiment Design

Based on the published research about the earthworm avoidance behavior test induced by metolachlor [7], the *Rac*-metolachlor and *S*-metolachlor exposure concentrations were set as 4, 16, and 64 µg/g of artificial soil. First, 10 g of artificial soil was contaminated with a certain amount of metolachlor acetone solution and thoroughly mixed until the acetone had completely evaporated. Afterward, 490 g of artificial soil was intensively mixed with the metolachlor-fortified soil. The soil without metolachlor contamination was set as the control. Finally, the treated soil samples were transferred into a 1 L glass beaker (Sichuan Shuniu Glass co., LTD, Chengdu, China) and adjusted to 35% moisture by adding deionized water. Each treatment was repeated six times (a total of 24 beakers). The acclimatized *E. fetida* were placed on moist filter papers (BKMAM, Hunan, China) for 24 h at 20 ± 1 °C and 80–85% humidity to void their gut contents. Ten gut-cleansed *E. fetida* were then placed in each beaker (5 g cow manure was evenly added to the soil surface every week). All the beakers were sealed with perforated plastic film. The beakers were incubated in an incubator at 20 ± 1 °C for 28 d under a 12/12 h light–dark cycle [8]. Twelve *E. fetida* were collected randomly from each treatment (two *E. fetida* for each beaker) after 3, 7, 14, and 28 d of exposure for determination of reactive oxygen species (ROS), SOD, MDA, and DNA damage (four earthworms for ROS, four earthworms for enzyme activity and MDA content, four earthworms for DNA damage). No *E. fetida* died during the exposure period. Soil (5 g) samples were collected at 1, 3, 7, 14, 21, and 28 d from each beaker, respectively, for residual determination of *Rac*-metolachlor and *S*-metolachlor. 

### 2.4. SOD, MDA, and ROS Determination

The collected *E. fetida* were first gut-cleaned for 24 h. After that, the *E. fetida* were weighed and homogenized in an ice-cold phosphate buffer solution (1:9 *w*/*v*, 50 mM, pH 7.5) and then centrifuged (Eppendorf centrifuge 5804R, Hamburg, Germany) at 10,000 rpm at 4 °C for 20 min. The supernatant was used to determine protein content, SOD activity, and MDA content. Coomassie brilliant blue staining method described by Bradford [9] was used to determine the total soluble protein concentration of the enzyme solution. The commercial kits were used to determine the SOD activity and MDA content according to the manufacturer’s instructions. The method of 2,7-dichlorofluorescin diacetate (DCFH-DA) fluorescence described Lawler et al. [10] was employed to determine the ROS level in *E. fetida*. First, the gut-cleaned *E. fetida* were homogenized in an ice-cold phosphate buffer solution (1:9 *w*/*v*, 100 mM, pH 7.5) and centrifuged at 3000 rpm at 4 °C for 10 min. The supernatant was centrifuged again at 20,000 rpm at 4 °C for 10 min and discarded the supernatant. The pellet was suspended immediately by adding 2 μM of DCFH-DA solution and then incubated at 37 °C for 30 min in the dark. The fluorescence intensity of the reaction solution was determined using a fluorescence spectrophotometer (F7000, Hitachi, Japan). The excitation and emission wavelengths were set to 488 and 522 nm, respectively. The final result was expressed as the fluorescence intensity per milligram of protein.

### 2.5. DNA Damage Analysis

The single-cell gel electrophoresis, also known as the comet assay [11], was used to determine DNA damage in *E. fetida* coelomocytes induced by metolachlor. The individual gut-cleaned *E. fetida* was firstly soaked in 1 mL of coelomic extracts (5% ethanol, 95% saline, 2.5 mg/mL EDTA, and 10 mg/mL guaiacol glyceryl ether) for 3 min [12]. The extruded coelomocytes suspensions were then transferred into a centrifuge and centrifuged at 3000 rpm for 10 min at 4 °C. The obtained coelomocytes precipitates were suspended using 1 mL of phosphate buffer (pH 7.4) for using the comet assay. Eighty microliters of 1.0% normal melting agarose (NMA) were dropped onto a glass slide. Twenty microliters of the coelomocytes suspensions were mixed quickly with 80 μL of 0.7% low melting agar (LMA) and pipetted onto NMA. On to the two layers of agarose, 80 μL LMA was added to fix them at 4 °C for 15 min. After solidification, the agarose gel was soaked in a fresh lysis buffer (2.5 M NaCl, 10 mM Tris,100 mM Na_2_EDTA, 1% sodium N-lauroylsarcosinate, 10% dimethyl sulfoxide, and 1% Triton X-100) at 4 °C for 10 min. The agarose gel was then soaked in an electrophoresis solution (300 mM NaOH, 1 mM Na_2_EDTA) at 4 °C for 20 min to unwind the DNA, followed by electrophoresis at 300 mA and 25 V for 25 min. Subsequently, the agarose gel was neutralized (0.4 M Tris-HCl, pH 7.5) thrice at 5 min intervals, after which the agarose gel was stained with ethidium bromide (2 mg/mL) for 10 min [13]. Finally, the agarose gel was observed by a fluorescence microscope (Olympus BX53, Japan) to obtain comet images. The comet images were analyzed using the CASP software (CaspLab, Poland) to obtain the relative parameters. In this study, the olive tail moment (OTM) value was used as an indicator of DNA damage [14]. 

### 2.6. Metolachlor Residual Determination in Soil 

The extraction and cleanup of metolachlor from soil samples were performed according to the method reported by Sun et al. [15]. Five grams of soil samples were put into a 50 mL polypropylene centrifuge tube. Subsequently, 5 mL of deionized water and 10 mL of acetonitrile were added to the centrifuge tube and vortexed at 2500 rpm/min for 3 min. Three grams of NaCl were then added to the centrifuge tube and vortexed again as described above. After which, the centrifuge tube was centrifuged at 3800 rpm/min for 5 min. The 5 mL of upper acetonitrile phase and 100 mg PSA were transferred into a 10 mL polypropylene centrifuge tube successively and vortexed for 5 min, following centrifuging at 4800 rpm/min for 3 min. The supernatant was filtered by a 0.22 μm nylon syringe for analysis. An Agilent 1100 high-performance liquid chromatography coupled with a DAD detector and a C18 column (250 m × 4.6 mm, 5 μm) was used to quantify the above samples. The mobile phase is acetonitrile (70%) and water (30%) with a 0.6 mL/min flow rate, a 25 °C column temperature, and a 200 nm detection wavelength. The injective volume was 20 μL. The fortified recoveries of metolachlor (1, 10, and 100 μg/g) were between 90.2% and 98.6%, and the relative standard deviations were less than 10%.

### 2.7. Statistical Analysis

The data were expressed as the mean±standard deviation and statistically analyzed using SPSS 19.0 (SPSS, Chicago, IL, USA). The data satisfy the hypotheses based on homoscedasticity and normality tests. Differences were determined by one-way ANOVA with Duncan’s post hoc analysis. The *p*-values < 0.05 and <0.01 were defined as statistically significant and extremely significant, respectively. The degradation of metolachlor in the soil was fitted by a first-order kinetic law: ln *C_t_* = ln *C_0_*^−kt^, where *C*_t_ denotes the concentration of the fungicide at time *t* and *C_0_* denotes the concentration of metolachlor at time zero, and *k* is the first-order degradation rate. The time for the degradation of 50% metolachlor was calculated as DT_50_ = ln2/*k*. 

## 3. Results

### 3.1. Effects of Rac- and S-metolachlor on ROS Levels in E. fetida

**Figure 1:**
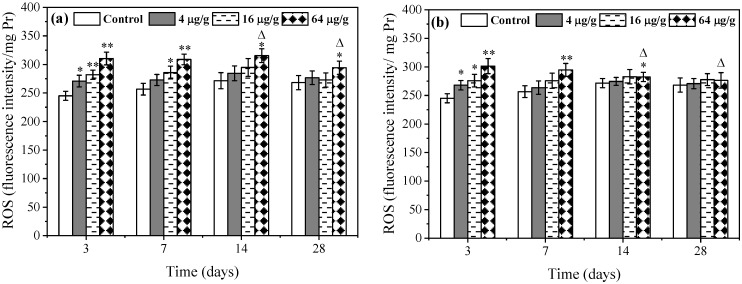
Effects of *Rac*- (**a**) and *S*-metolachlor (**b**) on ROS levels in *E. fetida*. * *p* < 0.05 and ** *p* < 0.01 indicate the statistical significance between the control group and treatment group at the same exposure time. “Δ” represents a significant difference between *Rac*- and *S*-metolachlor treatment groups at the same exposure concentration (*p* < 0.05).

As shown in Figure 1a, at day 3, ROS levels in all *Rac*-metolachlor treatment groups were significantly (*p* < 0.05) higher than those in the control treatment group, exhibiting a certain dose-effect relationship. In the highest *Rac*-metolachlor treatment group (64 μg/g), the ROS level increased by about 26.5% compared to the control treatment. As the exposure time lengthened, the ROS level at low (4 μg/g) and medium (16 μg/g) concentration treatments gradually recovered to the control level during the exposure period. However, the highest concentration of *Rac*-metolachlor still significantly induced ROS production, with a 9.7% increase observed after 28 days of exposure compared to the control. The changes of ROS in *E. fetida* caused by *S*-metolachlor were similar to that of *Rac*-metolachlor (Figure 1b). The difference was that ROS levels in all *S*-metolachlor treatments returned to control levels after 28 days of exposure, suggesting that *Rac*-metolachlor had a greater effect on ROS in *E. fetida* than *S*-metolachlor. In comparison between *Rac*- and *S*-metolachlor, the statistical results showed that there was no significant difference between 4 and 16 μg/g treatments during the 28 days of exposure. However, in the 64 μg/g treatment group, the ROS level was significantly (*p* < 0.05) higher under the stress of *Rac*-metolachlor than *S*-metolachlor on days 14 and 28.

### 3.2. Effects of Rac- and S-metolachlor on SOD Activity and MDA Content in E. fetida

**Figure 2:**
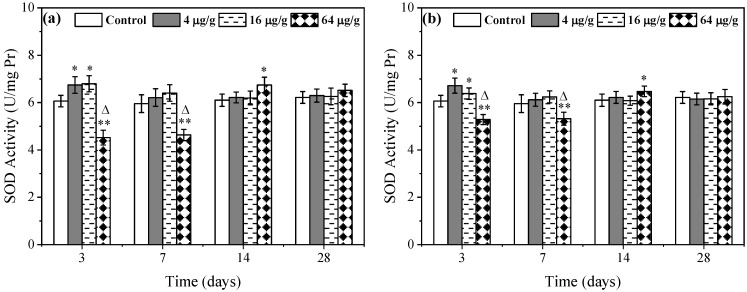
Effects of *Rac*- (**a**) and *S*-metolachlor (**b**) on SOD activity in *E. fetida*. * *p* < 0.05 and ** *p* < 0.01 indicate the statistical significance between the control group and treatment group at the same exposure time. “Δ” represents a significant difference between *Rac*- and *S*-metolachlor treatment groups at the same exposure concentration (*p* < 0.05).

The change of SOD activity in *E. fetida* caused by *Rac*- and *S*-metolachlor can be seen in Figure 2. As for 4 and 16 μg/g *Rac*-metolachlor treatments, the SOD activities were significantly (*p* < 0.05) stimulated at initial exposure (3 d) and then gradually returned to the control level. In the 64 μg/g *Rac*-metolachlor treatment, the SOD activities were significantly (*p* < 0.01) inhibited on the 3rd and 7th day, and the inhibition ratio reached 25.4% and 22.2% versus the control group, respectively. On day 14, the SOD activity was instead stimulated in *E. fetida* caused by 64 μg/g *Rac*-metolachlor, with a 10.5% stimulation ratio calculated (Figure 2a). As for the *S*-metolachlor treatment, the change of SOD activity in *E. fetida* was consistent with that of the *Rac*-metolachlor treatment during the exposure period (Figure 2b). There was no obvious difference was observed between *Rac*- and *S*-metolachlor in 4 and 16 μg/g treatments. However, the inhibition effect of *Rac*-metolachlor (64 μg/g) on SOD activity was significantly higher than that of the *S*-metolachlor treatment. The inhibitory response to *Rac*-metolachlor was 2.02 and 2.07 times greater than that of *S*-metolachlor on days 3 and 7, respectively. As exposure was extended, SOD activity in all *Rac*- and *S*-metolachlor treatments returned to the control level by day 28. 

**Figure 3:**
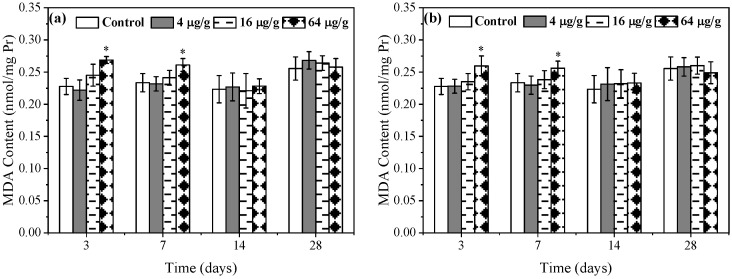
Effects of *Rac*- (**a**) and *S*-metolachlor (**b**) on MDA content in *E. fetida*. * *p* < 0.05 indicates the statistical significance between the control group and treatment group at the same exposure time.

The variation of MDA content is illustrated in Figure 3. The changes in MDA content in *E. fetida* induced by *Rac*- and *S*-metolachlor were consistent. Compared to the control, only the highest concentration (64 μg/g) induced the excessive production of MDA on days 3 and 7. After 14 days, there was no significant difference in MDA content between all the metolachlor treatments and controls. As for the comparison between *Rac*- and *S*-metolachlor, no significant difference was observed. 

### 3.3. Effects of Rac- and S-metolachlor on DNA Damage in E. fetida

**Figure 4:**
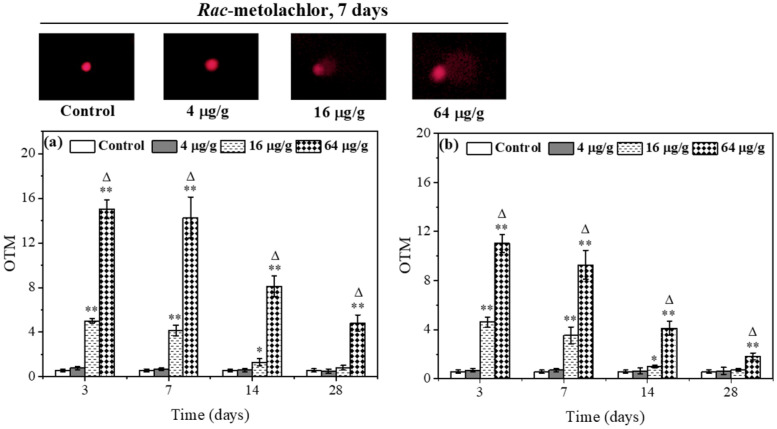
Effects of *Rac*- (**a**) and *S*-metolachlor (**b**) on DNA damage in *E. fetida*. * *p* < 0.05 and ** *p* < 0.01 indicate the statistical significance between the control group and treatment group at the same exposure time. “Δ” represents a significant difference between *Rac*- and *S*-metolachlor treatment groups at the same exposure concentration (*p* < 0.05).

In this study, the OTM value was used to indicate the degree of DNA damage. The larger the value, the more severe the DNA damage. As shown in Figure 4a, the OTM value in the 4 μg/g *Rac*-metolachlor treatment had no obvious difference compared to the control during the exposure period. In the 16 μg/g *Rac*-metolachlor treatment group, the OTM value was significantly higher than the control on days 3, 7, and 14, following recovered to the control lever on day 28. Under the stress of the 64 μg/g *Rac*-metolachlor, the OTM value was still significantly (*p* < 0.01) higher than the control during the 28 days of exposure, while a downward trend was observed with prolonged exposure. Figure 4b showed the variation of the OTM value for different concentrations of the *S*-metolachlor treatment, which was similar to the variation of the OTM value for the *Rac*-metolachlor treatment. In a comparison of *Rac*- and *S*-metolachlor, the OTM value of the 64 μg/g *Rac*-metolachlor treatment was always significantly (*p* < 0.05) higher than that of the 64 μg/g *S*-metolachlor treatment during the exposure period, indicating *Rac*-metolachlor induced more serious DNA damage to *E. fetida* than *S*-metolachlor.

### 3.4. Degradation of Rac- and S-metolachlor in Soil

**Figure 5:**
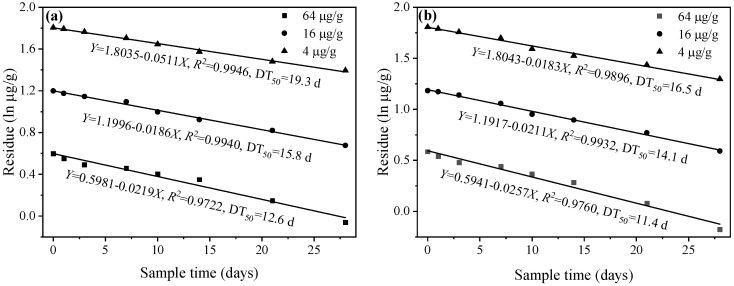
Degradation of *Rac*- (**a**) and *S*-metolachlor (**b**) in the soil.

In this study, the residual data were well fitted by the first-order kinetic equation (*R*^2^ > 0.97) and the result was shown in Figure 5. The calculated degradation half-lives (DT_50_) of 4, 16, and 64 μg/g *Rac*-metolachlor were 12.6, 15.8, and 19.3 d in the soil, respectively. As for *S*-metolachlor, its degradation half-lives were 11.4, 14.1, and 16.5 d at a concentration of 4, 16, and 64 μg/g, respectively. At the same treated concentration, the degradation half-live of *S*-metolachlor is generally shorter than that of *Rac*-metolachlor.

## 4. Discussion

In this study, we compared the enantioselective toxicity effects of *Rac*- and *S*-metolachlor on the earthworm *E. fetida*. Meanwhile, the degradation of two herbicides in the soil was also investigated. Results showed that *Rac*- and *S*-metolachlor produced different toxic effects on *E. fetida*. Additionally, the degradation rate was different for the two herbicides. 

As we know, ROS are chemically active oxygen-containing molecules in organisms and play an important role in cell signaling and homeostasis [16]. Typically, ROS can maintain dynamic equilibrium in organisms. However, ROS levels can increase dramatically and produce oxidative stress if the organisms suffered from environmental stress (e.g., xenobiotic pollutants) [17]. The result of this study showed that ROS had not excessively produced at 4 and 16 μg/g of both herbicide treatment groups except for the first 7 days of exposure. This indicated that the overproduction of ROS can be effectively scavenged by the antioxidant system in *E. fetida* [18]. With the increase in exposure concentration, the ROS level in the 64 μg/g *Rac*-metolachlor treatment was still higher than that of the control during the whole exposure period, suggesting that greater oxidative damage happened. The reason may be attributed to the overproduction of ROS beyond the scavenge ability of the antioxidant system in *E. fetida* [19]. Compared with the *Rac*-metolachlor treatment, 64 μg/g of *S*-metolachlor also induced the overproduction of ROS in *E. fetida*, while the inducement degree was significantly (*p* < 0.05) lower than the *Rac*-metolachlor treatment (Figure 1). This suggested that *Rac*-metolachlor induced stronger oxidative stress on *E. fetida*. ROS can be scavenged by some antioxidant enzymes, such as SOD. In organisms, SOD is an important peroxisomal enzyme that catalyzes the dismutation of superoxide radicals into hydrogen peroxide and oxygen [20]. SOD is often considered the first line of defense against ROS. In this study, the SOD activity significantly increased at 4 and 16 μg/g of both herbicides over 14 days of exposure (Figure 2), indicating SOD positively scavenged ROS induced by a lower concentration of both herbicides. Under the stress of the 64 μg/g *Rac*- and *S*-metolachlors, the SOD activity was significantly inhibited on days 3 and 7, suggesting the earthworms suffered from more stress damage. According to the ROS result and previous study, the reason can be due to the overproduction of ROS exceeding the ability of SOD and forming an inhibition or deactivation effect instead [18,21]. In comparison between the two herbicides, *Rac*-metolachlor showed a stronger effect on SOD activity than *S*-metolachlor. This result was consistent with the report by Xu et al. [7], who found that *Rac*-metolachlor had a greater effect on another antioxidant enzyme catalase in *E. fetida* compared to *S*-metolachlor. The different effects of *Rac*- and *S*-metolachlor on antioxidant enzyme activity may be attributed to both. One possible reason is that the stronger effect of *Rac*-metolachlor on SOD activity is probably due to stronger ROS production and SOD is more active as it has to scavenge more ROS. Another possible reason is that *S*-metolachlor has a lower enrichment content or faster metabolize rate in *E. fetida* than *Rac*-metolachlor [6,7]. Lipid peroxidation often occurs if earthworms are subjected to relatively severe oxidative stress. In this study, we determined the MDA content, which is a product of lipid peroxidation and usually indicates the degree of lipid peroxidation [22]. The result showed that only the highest concentration (64 μg/g) of both herbicides induced significant elevation of MDA in *E. fetida* on days 3 and 7 (Figure 3), which indicated that a high concentration of Rac- and S-metolachlor could induce lipid peroxidation in *E. fetida* in a short exposure time. Based on the results of ROS and SOD, this might be attributed to the highest exposure concentration, as the overproduction of ROS in *E. fetida* could not be effectively scavenged during the early exposure period, thereby producing lipid peroxidation [23]. The MDA content change in *Rac*- and *S*-metolachlor treatments had no obvious difference, indicating that both herbicides (within 64 μg/g) could not produce severe lipid peroxidation in *E. fetida* and had no significant enantioselective toxicity. As exposure was prolonged, the oxidative stress induced by both herbicides gradually returned to control levels after 14 days, indicating that the tested concentrations of Rac- and S-metolachlor did not cause irreversible oxidative damage to *E. fetida*. Based on the results of this study, the reduction in oxidative damage may be related to the degradation of pesticides and the adaptability of earthworms to the contaminated environment [18,23]. 

It is well-known that DNA damage is generally positively correlated with the degree of oxidative stress and lipid peroxidation in organisms. In DNA damage detection, a comet assay has been proven to be a rapid, simple, reliable, and sensitive method [24,25,26]. Therefore, in this study, we used a comet assay to determine DNA damage in *E. fetida* caused by *Rac*- and *S*-metolachlor. The results showed that there were no significant differences (*p* > 0.05) in the OTM values at the lowest concentration (4 μg/g) of both herbicides treatment compared with the control, while higher concentrations (16 and 64 μg/g) of both herbicides caused a significant (*p* < 0.01) increase in the OTM value (Figure 4). This indicated that *Rac*- and *S*-metolachlor could genetically impact *E. fetida* and this impaction was related to exposure concentration. According to our results and previous studies, the reason can be partially attributed to a higher concentration of metolachlor-induced overproduction of ROS in *E. fetida* which ultimately resulted in DNA damage [27,28]. As exposure was extended, the OTM values decreased for *Rac*- and *S*-metolachlor treatments, indicating that DNA damage was mitigated over time. This phenomenon could be attributed to some reasons as antioxidant enzyme protection, DNA self-repair, and pesticide degradation [23,29]. Based on the DNA damage classification criterion reported by Mitchelmore et al. [30], the experimental concentrations of *Rac*- and *S*-metolachlor only caused minimal and low DNA damage to coelomocytes of *E. fetida*. This is one of the reasons why DNA damage can be mitigated when the earthworms suffer from lower stress [12]. At the same exposure concentration and exposure time, the OTM values in the *Rac*- metolachlor treatment were significantly (*p* < 0.05) higher than those of *S*-metolachlor, indicating that *Rac*-metolachlor was more likely to produce genotoxicity to *E. fetida*. 

The dynamic results of the degradation of herbicides showed that the half-life of *S*-metolachlor was shorter than that of *Rac*-metolachlor in the soil (Figure 5), implying that the degradation process of both herbicides in the soil was enantioselective. The enantioselective difference had been reported as the result of soil microbial selectivity in the degradation of *Rac*- and *S*-metolachlor [31]. The degradation results for *Rac*- and *S*-metolachlor also support the fact that oxidative stress and DNA damage in *E. fetida* caused by both herbicides decreased over the time of exposure. In addition to the different degradation rates of the two herbicides in the soil, another reason may be that earthworms metabolize *S*-metolachlor more rapidly than *Rac*-metolachlor, resulting in less toxicity of *S*-metolachlor on *E. fetida* than *Rac*-metolachlor [7]. Based on the weed control effectiveness and toxicity differences of the two herbicides, a complete replacement of *Rac*-metolachlor with *S*-metolachlor in agricultural production is highly recommended. 

## 5. Conclusions

The difference in enantioselective toxicity of *Rac*- and *S*-metolachlor on *E. fetida* and their degradation are demonstrated in the present study. The results showed that the oxidative stress and DNA damage in *E. fetida* caused by higher concentrations (above 16 μg/g) of *Rac*- and *S*-metolachlor were significantly higher with respect to the control in early exposure. This toxicity is likely to decrease with longer exposure. Results of enantioselective oxidative stress and DNA damage caused by *Rac*- and *S*-metolachlor revealed that *Rac*-metolachlor had a greater effect than *S*-metolachlor. However, the detailed differences in enantioselective toxicity mechanisms still need to be investigated in the future. The results of degradation indicated that *S*-metolachlor degrades faster than *Rac*-metolachlor in the soil. Taken together, the results may provide important evidence for the scientific and appropriate application of *S*-metolachlor.

## Data Availability

The data that support the findings of this study are available from the corresponding author upon reasonable request.
